# Increased Arterial Diameters in the Posterior Cerebral Circulation in Men with Fabry Disease

**DOI:** 10.1371/journal.pone.0087054

**Published:** 2014-01-27

**Authors:** Nurcan Üçeyler, György A. Homola, Hans Guerrero González, Daniela Kramer, Christoph Wanner, Frank Weidemann, László Solymosi, Claudia Sommer

**Affiliations:** 1 Department of Neurology, University of Würzburg, Würzburg, Germany; 2 Department of Neuroradiology, University of Würzburg, Würzburg, Germany; 3 Würzburg Fabry Center for Interdisciplinary Therapy (FAZIT), University of Würzburg, Würzburg, Germany; Baylor Research Institute, United States of America

## Abstract

A high load of white matter lesions and enlarged basilar arteries have been shown in selected patients with Fabry disease, a disorder associated with an increased stroke risk. We studied a large cohort of patients with Fabry disease to differentially investigate white matter lesion load and cerebral artery diameters. We retrospectively analyzed cranial magnetic resonance imaging scans of 87 consecutive Fabry patients, 20 patients with ischemic stroke, and 36 controls. We determined the white matter lesion load applying the Fazekas score on fluid-attenuated inversion recovery sequences and measured the diameters of cerebral arteries on 3D-reconstructions of the time-of-flight-MR-angiography scans. Data of different Fabry patient subgroups (males – females; normal – impaired renal function) were compared with data of patients with stroke and controls. A history of stroke or transient ischemic attacks was present in 4/30 males (13%) and 5/57 (9%) females with Fabry disease, all in the anterior circulation. Only one man with Fabry disease showed confluent cerebral white matter lesions in the Fazekas score assessment (1%). Male Fabry patients had a larger basilar artery (p<0.01) and posterior cerebral artery diameter (p<0.05) compared to male controls. This was independent of disease severity as measured by renal function and did not lead to changes in arterial blood flow properties. A basilar artery diameter of >3.2 mm distinguished between men with Fabry disease and controls (sensitivity: 87%, specificity: 86%, p<0.001), but not from stroke patients. Enlarged arterial diameters of the posterior circulation are present only in men with Fabry disease independent of disease severity.

## Introduction

Fabry disease (FD) is an X-linked recessive lysosomal storage disorder with deficient activity of the enzyme α-galactosidase A (α-GAL). Enzyme deficiency is caused by mutations in the GLA-gene and leads to an accumulation of the glycosphingolipid globotriaosylceramide (Gb3) in kidneys, heart, and the nervous system [1]. The brain is mainly affected by a small vessel vasculopathy [2], [3] and white matter lesions (WML). FD is also a cause of stroke at young age [4]–[8].

Cerebral blood vessels of FD patients have been studied with Doppler sonography [9]–[11] and magnetic resonance imaging (MRI). In two studies of the same group of 25 FD patients cerebral artery diameters were measured using MRI with time-of-flight (TOF) MR-angiography (MRA) [12], [13]. The authors found an increased diameter of the basilar artery in FD patients compared to healthy controls and to stroke patients and suggested that the basilar artery diameter might be a diagnostic tool to detect FD patients among young patients with ischemic stroke [13]. Here we asked, whether these findings were true also in a large cohort of FD patients seen at our center, whether they were valid for women, and whether basilar artery diameter was dependent on disease severity as measured by renal function. We also assessed WML load and compared our results with non-FD patients with ischemic stroke and with controls.

## Patients and Methods

### Standard protocol approvals and patient consents

The study was approved by the Würzburg Medical School Ethics Committee. Written informed consent was obtained from all study participants at the time of examination.

### Subjects

In this retrospective study we analyzed data of 87 FD patients from the Würzburg University Hospital. These patients consecutively reported at the Würzburg Fabry Center for Interdisciplinary Therapy (FAZIT), and were examined at the Department of Neurology. FAZIT is a tertiary referral center where patients are seen from all over Germany mainly to confirm the diagnosis and to decide on the treatment. The MRI scans were performed at the Department of Neuroradiology as part of the routine work-up of Fabry patients. The patient group consisted of 30 men (median age 40 years, range 16–40 years) and 57 women (median age 45 years, range 16–73 years). Patients were included if FD was confirmed by α-GAL assay and genetically ascertained. Data on the peripheral nervous system [14] and on cerebral blood flow as measured by Doppler sonography [11] of some of these patients have been described elsewhere. More women with FD were included due to the higher number of men who carried cardiac defibrillators as contraindications for MRI scans.

We additionally analyzed data of 20 patients with cerebral ischemic stroke who were seen at our Department of Neurology between 2007 and 2012. This patient group consisted of 13 men (median age 58 years, range 49–89 years) and 7 women (median age 75 years, range 49–94years). Patients were included only, if cerebral ischemic stroke was confirmed by diffusion-weighted MRI, and if MRA had been performed.

Patients` data were compared with data of a group of 36 subjects with normal MRI scans who were examined at the Department of Neuroradiology, University of Würzburg from 2007 to 2012. These subjects received MRI scans e.g. to exclude hemorrhage or thrombosis in acute headache or structural lesions after head trauma or seizure. This control group consisted of 14 men (median age 44 years, range 26–72 years) and 22 women (median age 36, range 16–84 years).

### Clinical examination and laboratory tests

As part of the routine work-up all patients underwent neurological examination and laboratory tests including the assessment of the glomerular filtration rate (GFR) for renal function. Normal renal function was defined as GFR ≥60 ml/min/1.73 m^2^, reduced renal function was defined as GFR <60 ml/min/1.73 m^2^ [15]–[17].

### MRI scans

All patients and controls were investigated with a 3 tesla MRI scanner (Magnetom TIM Trio, Siemens, Germany). The following MRI sequences were performed (repetition time, TR; echo time TE): T1 (TR = 295 ms, TE = 4.67 ms, slice thickness: 5.0 mm, in plane resolution 0.7×0.6 mm), PD and T2 (TR: 2150 ms, TE: 11/101 ms, slice thickness: 5.0 mm, in plane resolution 0.9×0.9 mm), diffusion-weighted sequence (TR: 3300 ms, TE: 90 ms, slice thickness: 5.0 mm, in plane resolution 1.9×1.9 mm), time of flight (TOF) 3D MR-angiography (MRA) (TR: 24 ms, TE: 4.43 ms, slice thickness: 0.6 mm, in plane resolution 0.5×0.3 mm), fluid-attenuated inversion recovery (FLAIR) (TR: 7500 ms, TE: 134 ms, TI (inversion time): 2198 ms, FA (flip angle): 140°, slice thickness: 5.0 mm, in plane resolution 0.8×0.8 mm).

To determine WML load we applied the Fazekas score for each subject on the FLAIR sequences of the individual MRI scans. The Fazekas score ranges from zero to 3 with zero  =  no or a single punctuate white matter lesion, 1 =  multiple punctuate lesions, 2 = beginning confluence of lesions, 3 =  large confluent lesions [18].

Cerebral arteries were assessed using the 3D-reconstructions of the TOF-MRA scans. Vessel diameters were measured manually on identical sagittal planes by the same investigator and using MagicWeb VA60C_0212 Client Software (Visage Imaging). The following arteries were studied: common carotid artery (CCA), middle cerebral artery (MCA), anterior cerebral artery (ACA), posterior cerebral artery (PCA), and basilar artery (BA). Diameters were measured as follows: CCA in the middle of the vessel (relative to the most proximal and distal part of the CCA); MCA at the M1 segment; ACA at the A1 segment; PCA at the P1 segment; BA was measured in the middle of the vessel (relative to the most proximal and distal part of the BA). Data of paired arteries were averaged. All scans were assessed in the same time period and by an investigator unaware of the diagnoses and of the objectives of the study.

### Statistical analysis

IBM SPSS Statistics 20.0 software (Ehningen, Germany) was used for statistical analysis. Since data were not normally distributed the Mann-Whitney-U-test was applied for group comparisons. Receiver operating characteristic curves (ROC) were calculated for sensitivity and specificity analyses. P<0.05 was considered significant.

## Results

### Clinical and laboratory findings

Table 1 and Table S1 give baseline data of Fabry patients, patients with cerebral ischemic stroke, and of controls. There was no intergroup difference as for age between Fabry patients and controls. Patients with cerebral ischemic stroke naturally were older than patients with FD and controls. In 19 cases patients carried mutations that are classified as “classic” to date. In further 26 cases patients carried a known mutation. In eight cases intron polymorphisms were found. In one female patient with typical clinical presentation and positive family history, reduced alpha-galactosidase A levels were reported, however, genetic assessment has not been performed. In seven females multiple polymorphisms were found but with reduced alpha-galactosidase A activity and typical clinical presentation and/or positive family history. In the remaining 26 cases mutations in the alpha-galactosidase gene were detected that are currently not recognized as “classic”, however, alpha-galactosidase A activity, clinical presentation, and/or family history were typical for Fabry disease. Regarding central nervous system signs and symptoms, neurological examination was normal in 28/30 (93%) men and 54/57 (95%) women with FD. In the remaining five cases residual signs and symptoms were found after cerebral infarction such as unilateral facial hypoesthesia (1/87), unilateral drop in the arm and leg holding tests (2/87), and aphasia (2/5). Renal function was normal in the majority of men (73%) and women (88%) with FD.

**Table 1:** Characteristics of Fabry patients, patients with cerebral ischemic stroke, and of healthy controls.

	Fabry patients		Stroke patients		Controls			
	M	F	M	F	M	F		
N	30	57	13	7	14	22		
Age [years] (median, range)	40 (16–40)	45 (16–73)	58 (49–89)	75 (49–94)	44 (26–72)	36 (16–84)		
Time since diagnosis [years] (median, range)	3 (0–18)	3 (0–35)	acute	acute	n.a.	n.a.		
Cardiac involvement	21/30 (70%)	24/57 (42%)	n.a.	n.a.	n.a.	n.a.		
Renal involvement	8/30 (27%)	7/57 (12%)	n.a.	n.a.	n.a.	n.a.		
Pulmonary involvement	10/30 (33%)	4/57 (7%)	n.a.	n.a.	n.a.	n.a.		
ERT	23/30 (70%)	13/57 (23%)	n.a.	n.a.	n.a.	n.a.		
Time since ERT [years] (median, range)	4.5 (0.1–9.2)	4.9 (1.0–9.2)	n.a.	n.a.	n.a.	n.a.		
Fazekas 0	15	32	9	1	12	17		
Fazekas 1	12	23	2	3	2	4		
Fazekas 2	2	2	2	2	0	1		
Fazekas 3	1	0	0	1	0	0		
TIA/stroke	2/2	2/3	0/13	0/7	0/0	0/0		
No TIA/stroke	26	52	0	0	14	22		
							p-value: arterial diameters	
*TOF MRA (median; range)*							M Fabry vs	M Fabry vs
							M controls	M stroke
CCA [mm]	4.2 (3.4–6.5)	4.1 (2.9–5.5)	4.1 (3.2–5.3)	3.7(3.3–4.6)	4.8 (4.0–5.7)	3.9 (2.9–4.7)	n.s.	n.s.
MCA [mm]	2.4 (1.7–3.1)	2.3 (1.5–3.4)	2.1 (1.6–3.2)	2.2 (1.8–2.7)	2.3 (1.8–3.2)	2.2 (1.3–2.8)	n.s.	n.s.
ACA [mm]	1.9 (1.3–2.9)	1.7 (1–2.7)	1.5 (1.3–2.3)	1.2 (1.0–1.7)	1.6 (1.1–2.4)	1.7 (1–2.3)	n.s.	n.s.
PCA [mm]	1.9 (1.4–2.5)	1.9 (1.2–3.6)	1.7 (1.1–2.4)	1.7 (1.1–2.1)	1.5 (1.1–2.5)	1.7 (1–2.2)	<0.05	n.s.
BA [mm]	3.5 (2.7–4.4)	3.1 (2–5)	3.2 (2.6–4.2)	3.2 (1.3–3.6)	2.9 (2.3–5.5)	2.9 (1.2–3.6)	<0.001	n.s.

Abbreviations:

ACA: anterior carotid artery; BA: basilar artery; CCA: common carotid artery; ERT: enzyme replacement therapy; F: female; GFR: glomerular filtration rate; M: male; MCA: median cerebral artery; n.a.: not applicable; n.s.: not significant; PCA: posterior cerebral artery; TIA: transitory ischemic attack; TOF MRA: time-of-flight magnetic resonance angiography.

### TIA and strokes are frequent in FD

Table 1 gives data of cerebral events in Fabry patients. Two men (7%) and three women (5%) had experienced a cerebral stroke. TIA were reported by two men and women each (7%; 4%). All TIA and strokes were located in the anterior circulation.

### Confluent WML are rare in FD

Table 1 gives data of WML load in Fabry patients. Only one man (1%) of the entire Fabry population reached a Fazekas score of 3, and 2 men and women each had a score of 2, while the vast majority of men and women had no or only few single punctuate WML. This distribution was not different from that in patients with cerebral stroke or in controls. In none of the 87 FD cases a pulvinar sign was found.

### Men with FD have larger arterial diameters of the posterior circulation than male controls independent of disease severity

The diameter of the BA and the PCA was larger in FD patients (BA: median 4.1, range 2.9–6.5 mm; PCA: median 1.9, 1.2–3.6 mm) compared to controls (BA: median 2.9, range 1.2–5.5 mm, p<0.001; PCA: median 1.6, range 1.0–2.5 mm, p<0.01). The difference of BA and PCA diameters between FD and stroke patients (BA: median 3.2, range 1.3–4.2 mm; PCA: median 1.7, range 1.1–2.4 mm) was not significant. Also, the arterial diameters in the anterior circulation (CCA, MCA, ACA) did not differ between groups (data not shown).

Due to the X-chromosomal inheritance of FD the stratification of the patient groups for gender is crucial. This subgroup analysis revealed that only male Fabry patients had a larger BA diameter (p<0.001) and a larger PCA diameter (p<0.05) compared to male controls (Table 1). No such difference was found when comparing male FD patients with male stroke patients. The gender-wise comparison of data for the other study groups (stroke versus controls) also did not show intergroup differences and again in all study groups arterial diameters of the anterior circulation were similar irrespective of gender (Table 1).

We next separated the FD group for renal function as a marker of disease severity.

**Figure 1:**
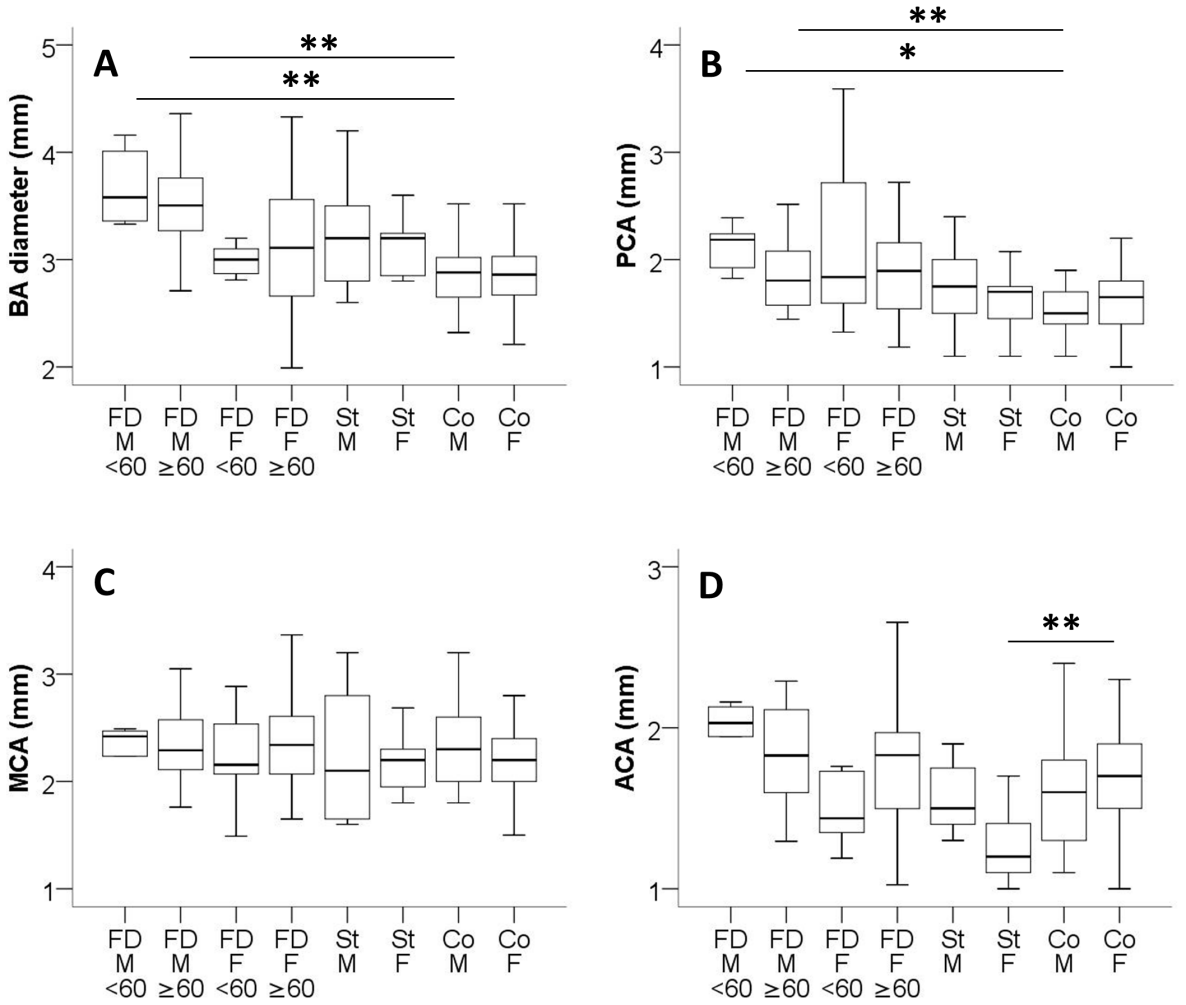
Cerebral artery diameters. Boxplots show the cerebral artery diameters of patients with Fabry disease (FD), cerebral ischemic stroke (St), and controls (Co) stratified for gender and renal function (A-D). (A) Male Fabry patients had a larger BA diameter independent of renal function. (B) Male Fabry patients had a larger PCA independent of renal function. (C) Subject groups did not differ in MCA diameters. (D) Female stroke patients had a smaller ACA diameter compared to female controls. The three study groups were compared gender-wise with each other. *p<0.05; **p<0.01. Patient numbers: Fabry male, GFR<60: n = 8; Fabry male, GFR≥60: n = 22; Fabry female, GFR<60: n = 7; Fabry female, GFR≥60: n = 50; Stroke male: n = 13; Stroke female: n = 7; Controls male: n = 14; Controls female: n = 22.

Unexpectedly, BA and PCA diameters of male FD patients with normal and impaired renal function did not differ (Table 2). Both subgroups had larger BA diameters (GFR ≥60 ml/min/1.73 m^2^: p<0.01; GFR <60 ml/min/1.73 m^2^: p<0.01) and PCA diameters (GFR ≥60 ml/min/1.73 m^2^: p<0.05; GFR <60 ml/min/1.73 m^2^: p<0.01) when compared with data of male controls (Table 1, 2; Fig. 1A, B). No such difference was found when comparing arterial diameters of patients with FD and stroke or of patients with stroke and controls (Table 1, 2; Fig. 1A, B) although subgroup analysis revealed a clear trend for the smallest BA diameters in controls followed by patients with stroke and by Fabry patients (Fig. 1A). Again, arterial diameters of the anterior circulation did not differ between subgroups (Table 1, 2; Fig. 1C-D). The only exception was the finding that female stroke patients had a smaller ACA diameter than female controls (p<0.001; Table 1; Fig. 1D). Arterial diameters did not correlate with age, α-GAL activity, or time since ERT (p>0.05). Also, no difference was found in vessel diameters of male and female patients with classic mutations compared to non-classic genetic alterations. None of the Fabry patients had dolichoectatic arteries.

**Table 2:** Characteristics of male and female Fabry patients with impaired renal function (advanced disease severity) and normal renal function (low disease severity).

	M GFR<60	M GFR≥60	F GFR<60	F GFR≥60
N	8	22	7	50
Age [years] (median, range)	46 (39–56)	34 (16–65)	57 (44–69)	42 (16–73)
Time since diagnosis [years] (median, range)	9 (0–18)	3 (0–16)	3 (0–10)	3 (0–35)
ERT	6	18	3	7
No ERT	2	4	4	43
Fazekas 0	4	11	4	28
Fazekas 1	4	8	3	19
Fazekas 2	0	2	0	2
Fazekas 3	0	1	0	0
TIA/stroke	0/2	2/0	1/0	1/3
No TIA/stroke	6	20	6	46
*TOF MRA (median; range):*				
CCA [mm]	4.2 (3.–5.2)	4.2 (3.4–6.5)	3.7 (3.5–4.3)	4.1 (2.9–5.5)
MCA [mm]	2.4 (1.7–2.5)	2.4 (1.7–3.1)	2.2 (1.5–2.9)	2.3 (1.7–3.4)
ACA [mm]	2 (1.4–2.2)	1.9 (1.3–2.9)	1.4 (1.2–1.8)	1.8 (1–2.7)
PCA [mm]	2.2 (1.8–2.4)	1.9 (1.4–2.5)	1.8 (1.3–3.6)	1.9 (1.2–2.7)
BA [mm]	3.6 (3.3–4.2)	3.5 (2.7–4.4)	3 (2.8–3.2)	3.1 (2–5)

Abbreviations:

ACA: anterior carotid artery; BA: basilar artery; CCA: common carotid artery; ERT: enzyme replacement therapy; F: female; GFR: glomerular filtration rate; M: male; MCA: median cerebral artery; TOF MRA: time-of-flight magnetic resonance angiography; PCA: posterior cerebral artery; TIA: transitory ischemic attack.

### Large BA diameter distinguishes men with FD from male controls

**Figure 2:**
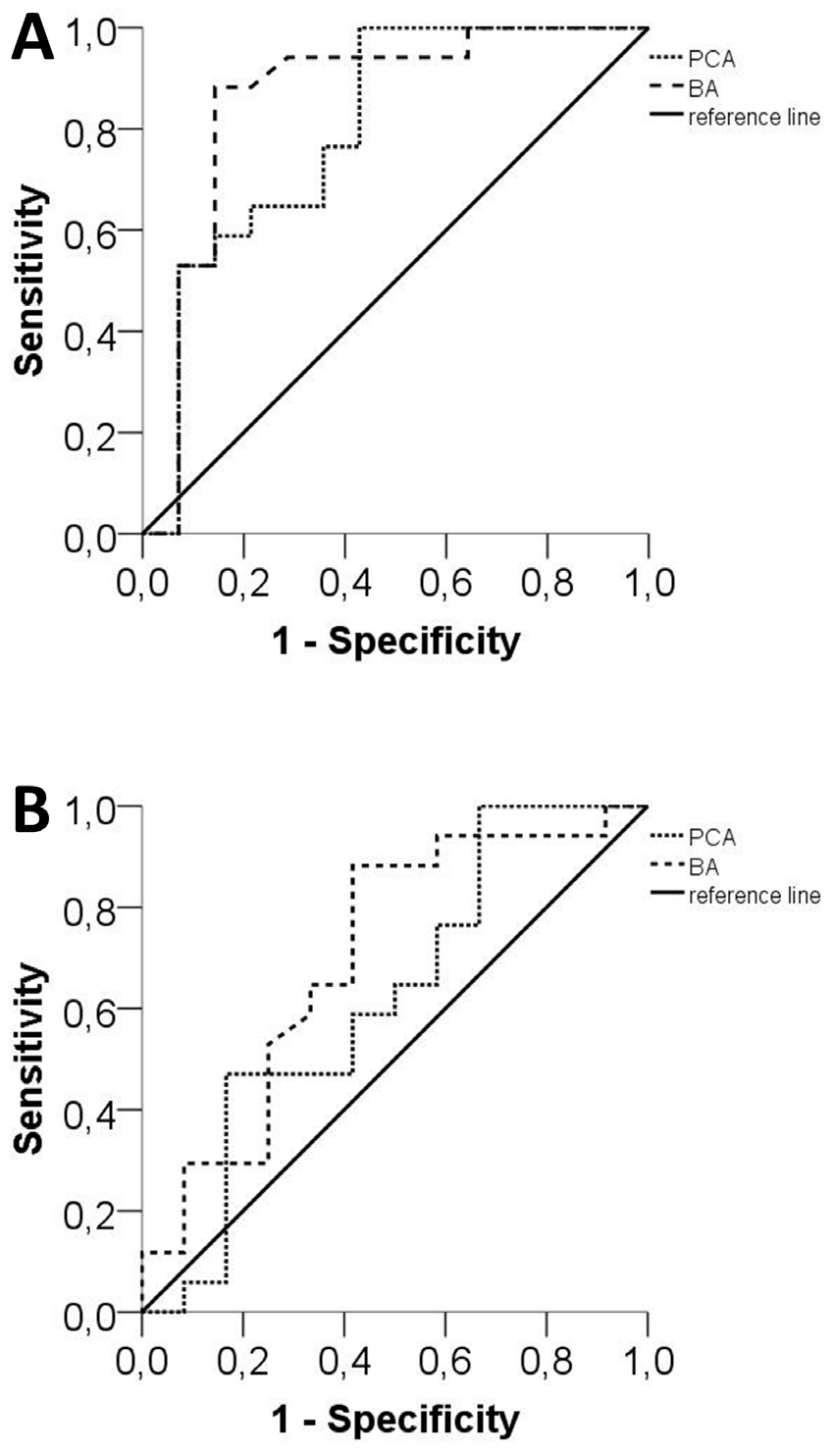
Sensitivity and specificity analysis of the basilar and posterior cerebral arteries. Graphs show receiver operating curves (ROC) for the sensitivity and specificity of BA and PCA diameters in male Fabry patients compared to male controls (A) and compared to male patients with cerebral ischemic stroke (B). With a cut-off value of 3.2 mm the BA diameter distinguished male Fabry patients from male controls with a sensitivity of 87% and specificity of 86% (A). The PCA diameter distinguished male Fabry patients from male controls at a cut-off value of 1.7 mm and with a sensitivity of 65% and a specificity of 79% (A). BA and the PCA diameters did not distinguish male Fabry patients from male stroke patients (B).

ROC analysis revealed that a BA diameter of >3.2 mm has a high sensitivity (87%) and specificity (86%) for distinguishing men with FD from male controls (area under the curve [AUC]: 0.859, p<0.001; Fig. 2A). Statistically, a PCA diameter of >1.7 mm also distinguished men with FD from male controls, however, with only a low sensitivity (65%) and specificity (79%; AUC: 0.798, p<0.05; Fig. 2A). BA and PCA diameters did not distinguish men with FD from male stroke patients (Fig. 2B). Also, BA and PCA diameters did not distinguish female FD patients from female stroke patients or female controls (p>0.05).

### Large BA diameter is not associated with increased incidence of TIA or stroke or high WML load

Nineteen male Fabry patients had a BA diameter of >3.2 mm; six of these patients also had a PCA of >1.7 mm. 10/19 patients had a Fazekas score of zero, 7/19 of 1, and 1/19 of 2. Only one man had a history of stroke in the anterior circulation, while 18/19 of these patients did not have a TIA or stroke medical history. GFR was normal in 14/19 patients. Of the four men with FD who reported TIA and stroke in their history only one had a Fazekas score of 3. BA and PCA diameters in these patients were below the cut-off values (i.e. were in the range of controls) calculated during ROC analysis.

### Large BA diameter does not change BA blood flow properties in FD

For seven male FD patients data of BA Doppler sonography data were available.[11] The median BA diameter of this subgroup of male patients was larger compared to male controls (p<0.001), however, BA blood flow parameters (mean, peak, and end-diastolic blood flow) did not differ from control values (data not shown).

## Discussion

In this large single-center study we show increased BA and PCA diameters only in men with FD compared to male controls and independent of disease severity as reflected by renal function. While we confirm a high number of TIA and strokes in Fabry patients, our data give no evidence for the predominance of severe structural cerebral changes in FD reflected by confluent cerebral WML.

Several case reports of dilated BA have been published [19]. Mitsias and Levine qualitatively reported two cases of FD with dilated BA; they also reviewed the literature for cerebral alterations in FD and found indications of dolichoectatic vertebrobasilar vessels in 51 historical patient reports [20]. In our cohort of 87 patients we did not find a case of dolichoectatic vertebrobasilar vessels, however, data cannot be directly compared due to different methodology. In a previous MRI study, mean BA and PCA diameters were enlarged in 25 male and female FD patients compared to healthy controls [12]. Data of the same patients were later compared with scan records of 26 selected young patients with cerebral ischemic stroke, and increased z-score values were reported for the BA diameter of FD patients compared to stroke patients suggesting a differentiating potential for the BA diameter [13]. Investigating a FD cohort more than three times bigger, we found larger BA and PCA diameters only in men with FD compared to male controls. In our cohort, BA diameter did not distinguish FD patients from patients with cerebral stroke.

Why male Fabry patients have a larger BA and PCA diameter than male controls and if a difference of 0.6 mm for the BA diameter, as was found here, is of biological relevance remains uncertain. It is of note that none of our FD patients had a cerebral ischemic stroke in the posterior circulation. Only one patient in the cohort of men with FD and enlarged BA diameter had a stroke in his medical history; the Fazekas score was zero or 1 in the vast majority of cases. Also, when regarding our previously published Doppler sonography data of those patients that had enlarged vessel diameters in the current study, we did not find a change in cerebral blood flow velocities [11]. Moore et al., using positron emission tomography (PET) and magnetic resonance arterial spin tagging (AST-MR), described cerebral hyperperfusion in male Fabry patients [21], [22]. However, how and if enlargements of proximal cerebral artery diameters and changes in regional cerebral blood flow measured by AST-MR are connected remains to be studied.

In our subgroup of 30 FD men, a BA diameter of >3.2 mm had a high sensitivity and specificity for distinguishing them from male controls. The results of the ROC analysis need to be interpreted with caution. To judge the clinical relevance of this potential diagnostic tool, a larger number of patients and a comparison with an already validated diagnostic tool would be desirable. We did not find a difference in BA diameter between our male FD patients and male stroke controls. One limitation of our study is that the group of patients with ischemic stroke was older than the group of Fabry patients. This is due to the fact that ischemic stroke is a disease of advanced age. However, since cerebral arterial diameters of stroke patients did not differ substantially from controls and since cerebral vessel diameters did not correlate with age we believe that our results were not influenced.

The pathogenesis of enlarged cerebral vessels is as yet incompletely understood. Factors that have been discussed are reduced sympathetic innervation of proximal cerebral arteries [6], [9], enhanced release of nitric oxide [23], and glycosphingolipid deposition in vascular smooth muscle cells [20]. The latter is supported by the few available autopsy reports [24], [25] and by the fact that enlarged BA have also been described in other lysosomal storage disorders like late onset Pompe disease [26]. In this context treatment with ERT might be effective in reducing glycosphingolipid deposition in the smooth muscle cells of vessel walls, however, no data are available to support this assumption so far. Another factor that potentially might influence vessel diameters in patients with FD is the level of blood carbon dioxide due to pulmonary involvement [27]; this parameter was not assess in our study, however, there were no clinical signs for severe pulmonary involvement in any of our patients.

There is no explanation, at present, why the posterior circulation is exclusively affected. In this context our finding that disease severity does not influence cerebral artery diameters is surprising. The X-chromosomal inheritance leads to a worse clinical manifestation in men and it is the men who have larger arterial diameters in the posterior circulation. However, although the peripheral nervous system deteriorates with advanced disease severity in FD [14] the brain seems to be protected. This is striking and further studies are needed to understand the underlying mechanisms.

In a previous study a high WML load on FLAIR images was reported in patients with FD [5]. Applying a similar semiquantitative scale, we could not confirm these data, and the lesion load was low in most of our patients, also in those with severe renal and cardiac involvement. In another study the quantitative assessment of WML on T2-weighted MRI scans in a cohort of 50 Fabry patients including children revealed that WML occurred in more than 70% of cases in the periventricular white matter region and that WML increased with age [28]. The individual WML was not reported and genders were not differentiated. The Fabry Outcome Survey (FOS) database reports WML in 38/84 patients, however without specification of methods and of disease severity [29], therefore a direct comparison with our data is not possible. However, it is conceivable that treatment has improved over time and that the burden of WML is therefore lower in more recent studies than in previous cohorts. It is also important to note that fewer patients with advanced FD can be investigated with MRI today due to the higher number of patients carrying defibrillators or pacemakers because of cardiac involvement [30]. This might induce a bias when cross-comparing data of earlier and current studies that might differ in the number of severely affected patients suitable for MRI. Another aspect that needs to be considered is the genotype of the patient population. Information on the exact and individual genetic alterations of the enrolled patients and if these mutations are recognized as classic or not to data is not provided in the majority of Fabry studies. Here we did not find differences in arterial diameters when comparing the different subtypes of genetic alterations in our cohort, however, one caveat is that these subgroups were small.

The pathophysiology of WML in FD is still elusive. The few available reports show lacunar infarctions and narrowing of small arterioles [24], [31] which might underlie WML, however, systematic data from larger studies are missing. So far no Fabry-specific pattern of WML spread has been found. The reports range from a focus in midbrain and brainstem [32] to predominantly in the carotid region [33]. The sensitivity and specificity of the pulvinar sign – as a potential indirect hint for disturbance of the posterior circulation – for Fabry disease is low. In two studies the presence of the pulvinar sign was investigated in men with Fabry disease comparing MRI scans with those of healthy controls [34], [35]. In both studies the sensitivity was <10%. Among studies investigating Fabry patients only with regard to a positive pulvinar sign data are conflicting (e.g. [6], [36]). Also, the biological meaning of WML is not well understood. Longitudinal studies investigating WML as potential risk factors for cerebral infarctions in FD have not been performed yet. Such studies would be needed to decide if high WML load requires special prophylactic precautions like anti-platelet drugs.

## Supporting Information

Table S1 **Characteristics of study population including treatment regimen with enzyme replacement therapy.** ^1^ Alpha-galactosidase alpha 0,2 mg/kg every second week ^2^ Alpha-galactosidase beta 1 mg/kg every second week ^3^ Alpha-galactosidase beta 1 mg/kg every second week, but with reduced dose during the period of product shortage ^4^ Alpha-galactosidase beta 1 mg/kg every second week, switched to alpha-galactosidase alpha during the period of product shortage Abbreviations: ERT: enzyme replacement therapy; F: female; M: male; cMRI: cranial magnetic resonance imaging.
(DOC)
