# Supplementary material for: Increased Arterial Diameters in the Posterior Cerebral Circulation in Men with Fabry Disease
Source: PLoS One. 2014 Jan 27;9(1):e87054. doi: 10.1371/journal.pone.0087054 (PMC3903616; doi:10.1371/journal.pone.0087054)
Supplement: Table S1 — Characteristics of study population including treatment regimen with enzyme replacement therapy. 1 Alpha-galactosidase alpha 0,2 mg/kg every second week 2 Alpha-galactosidase beta 1 mg/kg every second week 3 Alpha-galactosidase beta 1 mg/kg every second week, but with reduced dose during the period of product shortage 4 Alpha-galactosidase beta 1 mg/kg every second week, switched to alpha-galactosidase alpha during the period of product shortage Abbreviations: ERT: enzyme replacement therapy; F: female; M: male; cMRI: cranial magnetic resonance imaging. (DOC) [file pone.0087054.s001.doc]

**Table S1:** Characteristics of study population including treatment regimen with enzyme replacement therapy.

| **Pat. ID** | **Gender** | **Age**  **(years)** | **Time since diagnosis (years)** | **Enzyme activity (nmol/min/mg protein)** | **Genetic alteration (*classic)** | **Time since ERT until cMRI (years)** | **ERT** |
| --- | --- | --- | --- | --- | --- | --- | --- |
| 1 | M | 56 | 3.2 | 0.02 | Exon 6, position 10, transition G>A | 3.0 | Alpha-galactosidase alpha^1^ |
| 2 | M | 30 | 1.7 | 0.02 | Exon 7, position 1029, deletion TC | 1.5 | Alpha-galactosidase alpha^1^ |
| 3 | M | 25 | 6.9 | 0.03 | Exon 5, position 644, transition A>G* | 5.9 | Alpha-galactosidase alpha^1^ |
| 4 | M | 33 | 4.4 | 0.02 | Exon 7, position 1047, transition G>A* | 4.0 | Alpha-galactosidase beta^2^ |
| 5 | M | 41 | 1.4 | 0.06 | Exon 6, position 942, deletion G | 1.3 | Alpha-galactosidase beta^1^ |
| 6 | M | 44 | 8.0 | 0.02 | Exon 1, position 188, transition G>A | 6.1 | Alpha-galactosidase beta^3^ |
| 7 | M | 16 | 0.2 | 0.06 | Exon 3, position 416, transition G>A | 0.1 | Alpha-galactosidase beta^3^ |
| 8 | M | 49 | 9.1 | 0.02 | Exon 1, position 162, deletion T | 8.6 | Alpha-galactosidase beta^3^ |
| 9 | M | 22 | 3.4 | 0.04 | Exon 5, position 756-757, deletion T | 3.0 | Alpha-galactosidase alpha^4^ |
| 10 | M | 28 | 1.1 | 0.5 nmol/h/ml | Exon 7, position 169, transition C>T | 1.1 | Alpha-galactosidase beta^3^ |
| 11 | M | 65 | 1.4 | 10 nmol mU/mg | Exon 5, position 644, transition A>G | no ERT | no ERT |
| 12 | M | 44 | 8.4 | 0.02 | Exon 5, position 408, transition T>A | 7.1 | Alpha-galactosidase beta^3^ |
| 13 | M | 41 | 17.6 | 0.03 | Intron 3, IVS3+1 G>A | 8.6 | Alpha-galactosidase beta^3^ |
| 14 | M | 45 | 0.1 | 0.03 | Exon 7, position 1208, deletion AAG | 7.3 | Alpha-galactosidase beta^3^ |
| 15 | M | 26 | 0.1 | 0.04 | Exon 3, position 515, transition G>A | no ERT | no ERT |
| 16 | M | 34 | 0.3 | 0.28 nmol/h/mg | Intron 6, transition IVS6-10G>A; splice -site-mutation position 1000-10 G>A | 0.1 | Alpha-galactosidase alpha^1^ |
| 17 | M | 45 | 2.2 | 0.03 | Exon 7, position 1021, transition G>A | 2.0 | Alpha-galactosidase beta^2^ |
| 18 | M | 45 | 16.2 | 0.02 | Exon 7, position 1021, transition G>A | 7.0 | Alpha-galactosidase beta^3^ |
| 19 | M | 45 | 14.8 | 0.03 | Exon 3, position 305, transition G>A | 3.8 | Alpha-galactosidase beta^2^ |
| 20 | M | 26 | 4.4 | 0.02 | Exon 7, position 508, transition G>A | 3.8 | Alpha-galactosidase beta^2^ |
| 21 | M | 25 | 0.1 | 0.06 | Exon 6, position 934, transition C>T | no ERT | no ERT |
| 22 | M | 53 | 0.1 | 0.03 | Exon 1, position 611, transition G>A* | no ERT | no ERT |
| 23 | M | 20 | 0.1 | 0.06 | Exon 3, position 416, transition A>G | no ERT | no ERT |
| 24 | M | 34 | 2.6 | 0.02 | Exon 3, position 427, transition G>A | 2.5 | Replegal |
| 25 | M | 52 | 8.9 | 0.01 | Exon 3, position 494, transition A>T* | 8.1 | Alpha-galactosidase beta^2^ |
| 26 | M | 42 | 9.2 | 0.03 | Exon 7, position 494, transition A>T | 9.2 | Alpha-galactosidase alpha^4^ |
| 27 | M | 40 | 6.2 | 0.03 | Exon 7, position 494, transition A>T | 5.2 | Alpha-galactosidase alpha^1^ |
| 28 | M | 39 | 8.9 | 0.02 | Exon 7, position1196, transition G>A* | no ERT | no ERT |
| 29 | M | 45 | 5.9 | 0.02 | Exon 1, position 103, transition G>A | 5.0 | Alpha-galactosidase beta^2^ |
| 30 | M | 35 | 0.1 | 0.25 | Exon 3, position 427, transition G>A | no ERT | no ERT |
| 31 | F | 51 | 3.9 | 0.31 | Exon 6, position 993-994, insertion A | 2.8 | Alpha-galactosidase alpha^1^ |
| 32 | F | 26 | 0.1 | 0.3 | Exon 4, position 611, G>A* | no ERT | no ERT |
| 33 | F | 59 | 0.2 | 0.25 | Exon 7, position 1223, deletion A | no ERT | no ERT |
| 34 | F | 22 | 0.2 | 0.27 | Exon 7, position 1223, deletion A | no ERT | no ERT |
| 35 | F | 56 | 8.8 | 0.5 | Exon 5, position 644, transition A>G* | no ERT | no ERT |
| 36 | F | 47 | 35.4 | 0.09 | Exon 6, position 838, transition 838 | no ERT | no ERT |
| 37 | F | 41 | 6.9 | 0.28 | Exon 5, position 679, transition C>T* | 5.0 | Alpha-galactosidase alpha^1^ |
| 38 | F | 60 | 2.3 | 0.17 | Exon 6, position 994-994, insertion A | 1.0 | Alpha-galactosidase alpha^1^ |
| 39 | F | 51 | 3.7 | 0.11 | Exon 5, position 671, transition A>G* | 2.8 | Alpha-galactosidase alpha^4^ |
| 40 | F | 50 | 27.4 | 0.15 | Exon 7, position 1062-1076, deletion | 7.4 | Alpha-galactosidase beta^3^ |
| 41 | F | 61 | 0.7 | 0.16 | Exon 4, position 1221, deletion A | no ERT | no ERT |
| 42 | F | 19 | 6.1 | 0.25 | Exon 1, position 188, transition G>A | no ERT | no ERT |
| 43 | F | 43 | 8.2 | 0.12 | Exon 6, position 838, transition C>T | no ERT | no ERT |
| 44 | F | 49 | 0.2 | 0.21 | Exon 3, position 416, transition G>A | no ERT | no ERT |
| 45 | F | 49 | 4.8 | 0.3 | Intron 3 -10C>T,IVS4-16 A>G, IVS6-22 C>T | 4.3 | Alpha-galactosidase beta^3^ |
| 46 | F | 39 | 1.5 | 0.41 | Exon 3, position 427, transition G>A* | no ERT | no ERT |
| 47 | F | 31 | 6.2 | 7.2 | Exon 3, position 644, transition A>G | no ERT | no ERT |
| 48 | F | 22 | 3.8 | 0.29 | Exon 6, position 881, transition C>T | no ERT | no ERT |
| 49 | F | 45 | 3.2 | 0.22 | Exon 5, position 756-757, deletion T | no ERT | no ERT |
| 50 | F | 38 | 2.1 | 0.38 | Exon 3, position 427, transition G>A* | no ERT | no ERT |
| 51 | F | 21 | 0.6 | 0.15 | Exon 7, position 169, transition C>T | no ERT | no ERT |
| 52 | F | 37 | 0.4 | 0.29 | Intron 3, IVS2-81, -77 + IVS0-10C>T, IVS4-16A>G, IVS6-22 C>T | no ERT | no ERT |
| 53 | F | 67 | 7.6 | not determined | Exon 7, position 1208, deletion AAG | no ERT | no ERT |
| 54 | F | 62 | 2.2 | 0,44 | Exon 5, position 644, transition A>G | no ERT | no ERT |
| 55 | F | 57 | 2.6 | 0.18 | Exon 3, position 515, transition G>A | no ERT | no ERT |
| 56 | F | 31 | 2.6 | 0.2 | Exon 3, position 515, transition G>A | no ERT | no ERT |
| 57 | F | 20 | 6.3 | 0.8 mU/mg | Exon 6, position 937, transition G>T* | 5.4 | Alpha-galactosidase beta^3^ |
| 58 | F | 40 | 1.0 | 0.27 | Intron 3, IVS2-81.-77 + IVS0-10C>T, IVS4-16A>G, IVS6-22 C>T | no ERT | no ERT |
| 59 | F | 31 | 0.1 | 0.17 | Exon 3, position 1221, deletion A | no ERT | no ERT |
| 60 | F | 34 | 8.8 | 0.5 | Exon 7, position 560, transition A>G | 7.0 | Alpha-galactosidase beta^2^ |
| 61 | F | 48 | 13.7 | 0.41 | Exon 1, position 137, transition A>G* | no ERT | no ERT |
| 62 | F | 50 | 9.7 | 0.34 | Exon 7, position 1025, transition G>T | 9.2 | Alpha-galactosidase alpha^4^ |
| 63 | F | 54 | 0.1 | 0.4 | Intron 3 -10C>T | no ERT | no ERT |
| 64 | F | 69 | 0.2 | 0.23 | Exon 3, position 973, transition G>A | no ERT | no ERT |
| 65 | F | 49 | 2.9 | 0.5 | Exon 7, position 508, transition G>A | no ERT | no ERT |
| 66 | F | 35 | 0.1 | 0.42 | IVS0-10C>T, IVS4-16 A>G, IVS6-22 C>T | no ERT | no ERT |
| 67 | F | 24 | 0.6 | 0.4 | Exon 6, position 934, transition C>T | no ERT | no ERT |
| 68 | F | 51 | 2.7 | 0.43 | Exon 6, position 934, transition C>T | 1.7 | Alpha-galactosidase alpha^4^ |
| 69 | F | 24 | 0.6 | 0.26 | Exon 6, position 934, transition C>T | no ERT | no ERT |
| 70 | F | 18 | 1.0 | 0.18 | Exon 3, position 416, transition A>G | no ERT | no ERT |
| 71 | F | 47 | 0.1 | 0.29 | Exon 3, position 416, transition A>G | no ERT | no ERT |
| 72 | F | 53 | 9.7 | 0.43 | Exon 7, position 494, transition A>T | 9.2 | Alpha-galactosidase alpha^4^ |
| 73 | F | 43 | 4.0 | 0.22 | Exon 6, position 10, transition G>A | no ERT | no ERT |
| 74 | F | 73 | 5.0 | 0.16 | Exon 1, position 103, transition G>A | 4.4 | Alpha-galactosidase beta^3^ |
| 75 | F | 65 | 1.5 | 0.4 | Exon 3, position 427, transition G>A | no ERT | no ERT |
| 76 | F | 19 | 6.0 | 0.37 | Intron 2, IVS2+1 (G>A) | no ERT | no ERT |
| 77 | F | 16 | 5.5 | 0.18 | Exon 3, position 404, transition C>T* | no ERT | no ERT |
| 78 | F | 46 | 4.7 | 0.36 | Exon 3, position 404, transition C>T* | no ERT | no ERT |
| 79 | F | 32 | 0.1 | 0.29 | Exon 3, position 515, transition G>A* | no ERT | no ERT |
| 80 | F | 21 | 3.9 | 0.1 | Exon 3, position 404, transition C>T* | no ERT | no ERT |
| 81 | F | 47 | 3.9 | 0.16 | Exon 3, position 404, transition C>T* | no ERT | no ERT |
| 82 | F | 44 | 5.5 | 0.15 | Exon 4, position 612, transition G>A | 5.3 | Alpha-galactosidase beta^3^ |
| 83 | F | 40 | 0.1 | 0.5 | Exon 4, position 378, transition A>G | no ERT | no ERT |
| 84 | F | 58 | 7.8 | 0.1 | Exon 5, position 644, transition A>G* | no ERT | no ERT |
| 85 | F | 57 | 8.4 | <50% of normal value | not determined, but typical presentation and positive family history | no ERT | no ERT |
| 86 | F | 55 | 0.5 | 0.14 | Exon 5, position 1223, deletion A | no ERT | no ERT |
| 87 | F | 35 | 0.5 | 0.38 | IVS2-81.-77 + IVS0-10C>T, IVS4-16A>G, IVS6-22 C>T | no ERT | no ERT |
